# Genome Sequence of *Clostridium tyrobutyricum* ATCC 25755, a Butyric Acid-Overproducing Strain

**DOI:** 10.1128/genomeA.00308-13

**Published:** 2013-05-30

**Authors:** Ling Jiang, Liying Zhu, Xian Xu, Yanping Li, Shuang Li, He Huang

**Affiliations:** College of Food Science and Light Industry, Nanjing University of Technology, Nanjing, Chinaa; College of Biotechnology and Pharmaceutical Engineering, State Key Laboratory of Materials-Oriented Chemical Engineering, Nanjing University of Technology, Nanjing, Chinab; College of Sciences, Nanjing University of Technology, Nanjing, Chinac

## Abstract

*Clostridium tyrobutyricum* ATCC 25755 is an efficient producer of butyric acid. Here we report a 3.01-Mb assembly of its genome sequence and other useful information, including the coding sequences (CDSs) responsible for an alternative pathway leading to acetate synthesis as well as a series of membrane transport systems.

## GENOME ANNOUNCEMENT

Butyric acid, one of the four-carbon short-chain organic acids, is a specialty chemical with many applications in different industries, and currently there is an increasing interest in using it from microbial production as bio-based natural ingredients in foods, cosmetics, and pharmaceuticals (1). *Clostridium tyrobutyricum* ATCC 25755 is the most promising acidogenic bacterium used for the production of butyric acid (2–6). A highest butyric acid concentration of 86.9 g/liter has been achieved by our team with an adapted strain of *C. tyrobutyricum* ATCC 25755 (7). Moreover, genetic manipulation with this type strain, such as the inactivation of *ack* and *pta* genes, which encode enzymes associated with the acetate formation pathway, has been proved to significantly improve butyric acid production (8, 9). However, the formation of acetic acid was not completely eliminated in these mutants, suggesting the existence of other enzymes also leading to acetate synthesis. Therefore, investigation of the genetic information and characteristics of *C. tyrobutyricum* ATCC 25755 is desired. Genome-scale analysis has been proven useful for the metabolic engineering application (10).

Here we present the draft genome sequence of strain *C. tyrobutyricum* ATCC 25755, obtained using the Illumina Hiseq 2000 system, which was performed by Shanghai Majorbio Bio-Pharm Technology Co., Ltd. The reads were assembled with Velvet (1), and the sequence was annotated using the RAST annotation server (11). A library containing 300-bp inserts was constructed. Sequencing was performed based on the paired-end strategy of 101-bp reads to produce 1,667 Mb of filtered sequences, representing 553.82-fold coverage of the genome. The sequence of *C. tyrobutyricum* ATCC 25755 is 3,011,226 bases with a G+C content of 30.84%, which was assembled into 80 contigs and 67 scaffolds. They contain 3,040 open reading frames (ORFs), 46 tRNA genes, and 3 rRNA genes identified by Glimmer 3.02 (12), Genemark (13), tRNAscan-SE (14), and RNAmmer (15).

Inspiringly, acyl-coenzyme A (CoA):acetoacetyl-CoA transferase was detected among the gene products, which provided the first definitive evidence that there was an alternative pathway leading to acetate synthesis in acidogenic clostridia (8, 9). According to the genomic analysis, strain *C. tyrobutyricum* ATCC 25755 may have a powerful membrane transport capacity, including 20 protein-coding sequences (CDSs) involved in the phosphotransferase systems and the corresponding regulatory system analogs (e.g., Hpr), 106 CDSs involved in ATP-binding cassette (ABC)-type transporters, and 1 involved in P-type ATPases, as well as many uncharacterized transporters. They may play important roles in the production of butyric acid with high productivity (6, 7). Additionally, several putative enzymes were also annotated in the *C. tyrobutyricum* ATCC 25755 genome, such as glycosyltransferase, aspartate transaminase, sugar phosphate isomerase, amino acid permease, glucokinase, intracellular protease/amidase, amidohydrolase, helicase, and some regulatory proteins. Further studies will be performed to confirm their functions, and a complete genome sequence will be included in the future to reveal the unique molecular characteristics of strain *C. tyrobutyricum* ATCC 25755.

### Nucleotide sequence accession numbers.

This whole genome shotgun project has been deposited at DDBJ/EMBL/GenBank under accession no. APMH00000000. The version described in this paper is the first version, with accession no. APMH01000000.
